# Discovery of *ETS1* as a New Gene Predisposing to Dilated Cardiomyopathy

**DOI:** 10.3390/diagnostics15162031

**Published:** 2025-08-13

**Authors:** Zun-Ping Ke, Jia-Ning Gu, Chen-Xi Yang, Xue-Lin Li, Su Zou, Yi-Zhe Bian, Ying-Jia Xu, Yi-Qing Yang

**Affiliations:** 1 Department of Geriatrics, Shanghai Fifth People′s Hospital, Fudan University, Shanghai 200240, China; xuelin8a@163.com; 2 Department of Cardiology, Shanghai Fifth People′s Hospital, Fudan University, Shanghai 200240, China; jngu20@fudan.edu.cn (J.-N.G.); cxyang21@m.fudan.edu.cn (C.-X.Y.); zou_su@163.com (S.Z.); voxens@outlook.com (Y.-Z.B.); xuyingjia@5thhospital.com (Y.-J.X.); 3 Department of Central Laboratory, Shanghai Fifth People′s Hospital, Fudan University, Shanghai 200240, China; 4 Department of Cardiovascular Research Laboratory, Shanghai Fifth People′s Hospital, Fudan University, Shanghai 200240, China

**Keywords:** cardiomyopathy, medical genetics, functional genomics, transcription factor, ETS1, reporter gene assay

## Abstract

**Background/Objectives:** Dilated cardiomyopathy (DCM), defined as dilation and contractile dysfunction of the left or both cardiac ventricles, remains the most common category of primary myocardial disease worldwide. It is the most prevalent cause of chronic heart failure and the most common indication for cardiac transplantation in young subjects. Accumulating evidence increasingly highlights the substantial genetic defects underlying DCM. Nevertheless, the genetic ingredients accountable for DCM in a major percentage of patients remain indefinite. **Methods:** A multigenerational pedigree suffering from DCM and a total of 276 healthy volunteers employed as controls were recruited from the Chinese Han-ethnicity population. A whole-exome sequencing (WES) assay followed by a Sanger sequencing analysis of the genomic DNAs from the available family members was implemented. Functional characterization of the identified genetic variant was completed by dual-luciferase analysis. **Results:** A new heterozygous variation in the *ETS1* (erythroblast transformation-specific 1) gene, NM_005238.4:c.447T>G;p.(Tyr149*), was identified by WES and validated by Sanger sequencing analysis to co-segregate with DCM in the whole DCM family. This nonsense *ETS1* variant was not found in 276 control subjects. Functional examination elucidated that Tyr149*-mutant ETS1 lost the ability to transactivate its downstream target genes *CLDN5* (claudin 5) and *ALK1* (activin receptor-like kinase 1), two genes crucial for cardiovascular embryonic development and postnatal structural remodeling. **Conclusions:** The present investigation reveals *ETS1* as a new gene predisposed to human DCM and indicates *ETS1* haploinsufficiency as an alternative molecular pathogenesis underlying DCM, providing a potential molecular target for genetic counseling and early diagnosis as well as personalized prophylaxis of DCM.

## 1. Introduction

Dilated cardiomyopathy (DCM), which is clinically characterized by progressive enlargement and systolic dysfunction of the left or both cardiac ventricles without a known secondary cause such as valvular heart disease, coronary artery disease, metabolic derangement, essential hypertension, infiltrative disease, viral infection, nutrient deficiency, exposure of medications and toxins, autoimmune disease, or any others, represents the most prevalent category of primary myocardial disorder in humans worldwide, occurring in up to 0.45% of the general population [1,2,3]. There exists a slight woman preponderance in the prevalence of DCM, with an estimated male-to-female ratio of 3:4 [2]. DCM is associated with substantially enhanced risks for many detrimental clinical sequelae, encompassing ischemic stroke/thromboembolic complications [4,5,6], cardiac conduction block/abnormality [7,8,9], atrial fibrillation [10,11,12], chronic congestive heart failure [13,14,15,16,17,18], fatal/malignant ventricular dysrhythmias [19,20,21], and even cardiac demise [22,23,24]. In fact, in young individuals, DCM is the most prevalent cause of congestive heart failure and the most frequent indication for cardiac transplantation globally [25]. According to an observational study, approximately 26% of children with DCM experience either cardiac death or the necessity for a cardiac organ transplant within one year after initial diagnosis of DCM, with an additional increase of 1% per year thereafter [26]. Additionally, in adults aged 20–60 years, DCM is also the most common etiology of congestive/chronic heart failure and cardiac death, and the leading reason for heart transplant worldwide [25]. According to recent long-term studies of patients affected with nonischemic DCM, about 50% suffered from a composite of unplanned cardiovascular hospitalization, life-threatening/nonfatal arrhythmia, or cardiovascular demise over 12 years [27], and roughly 17% underwent cardiac death, heart transplant, or implantation of a ventricular assist device over 8 years [28]. In the United States alone, each year DCM causes approximately 46,000 hospitalizations and 10,000 deaths [29]. Consequently, DCM leads to substantial morbidity and mortality, and confers a tremendous socioeconomic burden on humans, underscoring the pressing need to unravel the causes underpinning DCM.

The etiology of DCM is extremely complex and highly diverse, and both exogenous/nonheritable risk factors and endogenous/genetic defectives may give rise to DCM [1,2,30,31]. Well-known acquired risk factors contributing to DCM include toxic chemicals (such as alcohol, clozapine, anabolic/adrenergic steroids, amphetamines, and anthracyclines), hemodynamic/mechanical factors (such as regurgitant/stenotic heart valve disease, anemia, athleticism/exercise, atrial tachycardias, premature ventricular beats, myocardial infarction/coronary artery disease, and pregnancy), inflammatory/infiltrative factors (such as immune myocarditis, pathogen myocarditis, and cardiac amyloidosis), endocrine/metabolic factors (such as diabetes mellitus, Cushing disease, acromegaly, pheochromocytoma, hypothyroidism, hyperlipidemia, and hyperthyroidism), hypertension, obesity, nutritional deficiency, obstructive sleep apnea, chronic kidney disease, hepatic cirrhosis, neuromuscular disease, severe sepsis, thoracic radiotherapy, and smoking [30,31]. However, there is a fast-growing body of data convincingly suggesting the pivotal effect of heritable determinants on the development of DCM, especially on the development of familial DCM, which is defined as the presence of ≥2 relatives with DCM or the presence of one relative with DCM along with sudden cardiac death before the age of 35 years [1,2]. It is reported that around 30% of DCM is familial [31], and in up to 50% of DCM patients, DCM is transmitted in an autosomal-dominant mode, though less common patterns of transmission, encompassing X-linked recessive inheritance and autosomal-recessive inheritance along with mitochondrial inheritance, have also been reported in some DCM patients [32]. At present, DCM-causing variations in >250 genes implicated in many distinct cellular processes have been identified, which include genes involved in force generation (sarcomere, Z-disk, sarcolemma, and intercalated disc proteins), cellular architecture (cytoskeleton, nuclear envelope, and desmosome proteins), gene expression (transcription factors and RNA-binding proteins), energy regulation (mitochondria), electrolyte homeostasis (ion channels and gap junction channels), and signaling transduction [1,2,33,34,35,36,37,38,39,40,41,42,43,44,45,46,47,48,49,50,51,52,53,54,55,56,57,58,59,60,61]. Notably, genetic defects responsible for DCM are discovered much more commonly in pediatric cases (54%) than in adult patients (27%) [2] Nevertheless, these known genetic determinants account for 25–50% of all DCM cases, strongly indicating that additional genetic components responsible for DCM remain to be identified [1,2,25].

## 2. Materials and Methods

### 2.1. Ethics

The current human research project was implemented in complete compliance with the tenets outlined in the Helsinki Declaration issued by the World Medical Association. The institutional Medical Ethics Committee of Shanghai Fifth People′s Hospital affiliated to Fudan University approved the research protocols (ethical approval no.: 2020-011; ethical approval date: 13 January 2021) that were applied to the current human research. Prior to commencing recruitment for the current research, all candidate research participants or their legal guardians signed informed consent forms to fulfill clinical and genetic analyses.

### 2.2. Recruitment and Basic Clinical Characterization of Study Participants

For the present study, a 42-member pedigree across five generations affected with DCM was discovered from the Han-race population in China (arbitrarily termed as Family DCM-101), from which available pedigree members were recruited. As control subjects, a cohort of 276 unrelated healthy volunteers with no family history of heart disease was enlisted from the same population in the same geographical area. All research participants underwent a comprehensive clinical assessment, including a thorough review of anamnestic personal and familial histories as well as medical history, an elaborate physical examination, transthoracic echocardiographic imaging, and standard 12-lead electrocardiography, along with routine laboratory tests. All DCM patients underwent a maximal exercise performance test. However, standard cardiac magnetic resonance imaging, coronary computed tomography angiography, and an endomyocardial biopsy were performed only when strongly indicated. As previously described [37], DCM was diagnosed by the existence of a left ventricular end-diastolic diameter >117% of the predicted value adjusted for age and body surface area and fractional shortening < 25% or ejection fraction <45% under the conditions of no obstructive coronary artery disease, sustained arterial hypertension, cardiac valve disorder, documented myocarditis, or systemic disorders. A total of 3 mL of peripheral venous blood samples was collected from each study individual and stored in a collecting tube containing an anticoagulant (acid-citrate dextrose) in a low-temperature (−80 °C) refrigerator. Extraction of genomic DNA from research participants’ whole blood leucocytes was conducted utilizing the QIAamp^®^ DNA Blood Mini Kit (Cat. No. 51106; QIAGEN GmbH, Hilden, Germany).

### 2.3. Genetic Analysis of Research Participants

As described previously [62,63,64,65,66], a whole-exome sequencing (WES) assay was performed on genomic DNA samples from selected affected and unaffected members of Family DCM-101 suffering from DCM. Briefly, a sample of 5 μg of genomic DNA from a family member chosen for WES was randomly sheared via the Covaris^®^ S2 Ultrasonicator (Covaris, Woburn, MA, USA) to generate various sizes of DNA fragments with a major length distribution of 100 to 500 bps, which were ligated with sequencing adapters and captured using the SureSelect^XT^ Human All Exon V6 Kit (Cat. No. 5190-8864; Agilent Technologies, Santa Clara, CA, USA) to obtain a whole-exome library. The exomic DNA library was sequenced on the Illumina HiSeq^®^ 4000 platform (Illumina, San Diego, CA, USA). Bioinformatic analyses of the sequence datasets produced by WES were implemented as narrated elsewhere [62,63,64,65,66]. In brief, alignment of read data to the referential human genome (build GRCh37/hg19) was implemented after removing low-quality reads by employing the Burrows–Wheeler Aligner (BWA; version 0.7.17) software package [67]. Thereafter, duplicated reads were ruled out, and the Genome Analysis Toolkit (GATK; version 3.8.1.0) software [68] was applied to call sequence variations (small insertions and deletions as well as single-nucleotide polymorphisms) at the targeted regions of an individual genome. All genetic variants were annotated by the ANNOVAR (version 2015) program [69]. Nonsynonymous variations, along with splicing donor/acceptor variations, were subject to further examination, encompassing a Sanger sequencing examination of a gene with a deleterious variation along with a segregation assessment among all the family members from Family DCM-101. Once a gene harboring a DCM-causing mutation was discovered by WES assay in Family DCM-101, a Sanger sequencing assay of the same gene (including the entire coding exons as well as all splicing junction sites) was performed in 276 unrelated control persons. Additionally, for each identified genetic variation, the databases of gnomAD (http://gnomad-sg.org/, accessed on 20 June 2025) and dbSNP (https://www.ncbi.nlm.nih.gov/, accessed on 20 June 2025) were consulted to verify its novelty.

### 2.4. Recombination of Gene Expression Plasmids

As depicted previously [70,71,72], total mRNAs were purified from human myocardial tissue specimens, from which cDNAs were yielded by routine reverse transcription of total mRNAs. A 1700-bp cDNA fragment containing the full-length ORF as well as partial 5′ and 3′ untranslated regions of the wild-type human erythroblast transformation-specific 1 (*ETS1*) gene (https://www.ncbi.nlm.nih.gov/nuccore/NM_005238.4, accessed on 16 July 2024) was amplified from purified human heart cDNA by polymerase chain reaction (PCR) using the Platinum^TM^ SuperFi II DNA Polymerase (Cat. No. 12361050; Invitrogen, Carlsbad, CA, USA) with the pair primers of 5′-GTCGCTAGCGAGATCGAGAGCGAACGAGG-3′ and 5′-GTCGCGGCCGCACCAACACGGCTGTCCTTGG-3′. The generated *ETS1* cDNA was purified with the QIAEX II Gel Extraction Kit (Cat. No. 20051; QIAGEN GmbH, Hilden, Germany). The purified *ETS1* cDNA and eukaryotic expression plasmid pCI-neo (Cat. No. E1841; Promega, Madison, WI, USA) were doubly cut with restriction endonucleases *Nhe*I (Cat. No. ER0971; Thermo Scientific, Waltham, MA, USA) and *Not*I (Cat. No. ER0595; Thermo Scientific, Waltham, MA, USA), purified employing the GeneJET™ Gel Extraction Kit (Cat. No. K0692; Thermo Scientific, Waltham, MA, USA), and ligated by T_4_ DNA ligase (Cat. No. 15224041; Invitrogen, Waltham, MA, USA) to create the wild-type ETS1-pCI plasmid ETS1-pCI. With the wild-type ETS1-pCI plasmid used as a PCR template, the Tyr149*-mutant ETS1-pCI expression plasmid was generated through site-targeted mutagenesis utilizing the GENEART^®^ Site-Directed Mutagenesis System (Cat. No. K0692; A13282; Invitrogen, Waltham, MA, USA) with the complementary oligonucleotide primer pairs of 5′-AGTCAACCCAGCCTAGCCAGAATCCCGCTAT-3′ and 5′-ATAGCGGGATTCTGGCTAGGCTGGGTTGACT-3′ and was validated by Sanger sequencing assay. In addition, a 1101-bp promoter fragment of the human claudin 5 (*CLDN5*) gene (GenBank accession no.: NC_000011.10) was PCR-amplified from human genomic DNA with the DreamTaq™ DNA Polymerase (Cat. No. EP1702; Thermo Scientific, Waltham, MA, USA) and a *CLDN5*-specific pair of primers of 5′-CTAGGTACCGGAGGCCGCAGCAGTCATCC-3′ and 5′-CTAAAGCTTCTCAATCTTCACAGGGGCTG-3′. The produced *CLDN5* promoter DNA as well as the reporter plasmid of pGL3-Basic (Cat. No. E1751; Promega, Madison, WI, USA) was doubly cut with the restriction enzymes *Kpn*I (Cat. No. ER0521; Thermo Scientific, Waltham, MA, USA) and *Hind*III (Cat. No. ER0501; Thermo Scientific, Waltham, MA, USA), separated by agarose gel electrophoresis, extracted with the GenElute™ Gel Extraction Kit (Cat. No. NA1111; Sigma-Aldrich, St. Louis, MO, USA), and ligated with T4 DNA ligase (Cat. No. 15224041; Invitrogen, Waltham, MA, USA) to construct the CLDN5-luciferase (CLDN5-luc) reporter vector, which expresses firefly luciferase. Similarly, a 1690-bp promoter fragment (from −221 to −1910 upstream of the transcriptional initial site) of the human activin receptor-like kinase 1 (*ALK1*) gene (https://www.ncbi.nlm.nih.gov/nuccore/NC_000012.12?from=51906944&to=51923361&report=genbank, accessed on 16 July 2024) was PCR-amplified from human genomic DNA with the DreamTaq™ DNA Polymerase (Cat. No. EP1702; Thermo Scientific, Waltham, MA, USA) as well as an *ALK1*-specific pair of primers of 5′-GATGGTACCGACTCTCGCTTCTCAGGAC-3′ and 5′-GATAAGCTTCGCACCCGGGCCGGGCCTCC-3′. The yielded *ALK1* promoter DNA as well as the reporter plasmid of pGL3-Basic (Cat. No. E1751; Promega, Madison, WI, USA) were digested with the restriction enzymes *Kpn*I (Cat. No. ER0521; Thermo Scientific, Waltham, MA, USA) and *Hind*III (Cat. No. ER0501; Thermo Scientific, Waltham, MA, USA), separated by agarose gel electrophoresis, extracted with the GenElute™ Gel Extraction Kit (Cat. No. NA1111; Sigma-Aldrich, St. Louis, MO, USA), and ligated with T4 DNA ligase (Cat. No. 15224041; Invitrogen, Waltham, MA, USA) to construct the ALK1-luciferase (ALK1-luc) reporter vector, which expresses firefly luciferase. All the final recombinant expression plasmids were subject to confirmation by Sanger sequencing analysis.

### 2.5. Cell Transfection and Dual Reporter Gene Analysis

As narrated previously [37,73,74], HeLa cells were seeded into a Nunc™ 24-well microplate/multidish (Cat. No. 144530; Thermo Fisher Scientific, Rochester, NY, USA) at a density of 1 × 10^5^ cells/well, and routinely cultivated for 24 h before transient transfection with appropriate amounts of various expression plasmids using the Lipofectamine™ 3000 Transfection Reagent (Cat. No. L3000015; Invitrogen; Waltham, MA, USA). Specifically, HeLa cells were transiently transfected with the same amount (200 ng) of empty pCI, or wild-type ETS1-pCI, or Tyr149*-mutant ETS1-pCI, or 100 ng of empty pCI plus 100 ng of wild-type ETS1-pCI, or 100 ng of wild-type ETS1-pCI plus 100 ng of Tyr149*-mutant ETS1-pCI, together with 10 ng of pGL4.75 (Cat. No. E6931; Promega, Madison, WI, USA) and 1000 ng of CLDN5-luc or ALK1-luc. As an internal control, the pGL4.75 plasmid (Cat. No. E6931; Promega, Madison, WI, USA) expressing Renilla luciferase was co-transfected to balance transfection efficiency. HeLa cells were collected 36 h post-transfection, lysed, and the promoter-driven luciferase activities were quantitatively detected as described previously [37]. The promoter activity was measured as the ratio of firefly/Renilla luciferase luminescence intensity. Each recombinant expression plasmid, along with an internal control plasmid, was transiently transfected and detected three times in triplicate. Results are expressed as means of relative luciferase activity from three independent transfection experiments and standard deviations (SDs).

### 2.6. Statistical Analysis

Continuous data of promoter activities are presented as mean ± SD. All data were analyzed by using Student’s unpaired *t*-test for pairwise comparison or analysis of variance (ANOVA) followed by Tukey–Kramer HSD post hoc test for multi-group analysis. Differences were considered to be statistically significant at two-tailed *p*-values of <0.05.

## 3. Results

### 3.1. Demographic and Phenotypic Characteristics of the Recruited Family Affected with DCM

**Figure 1:**
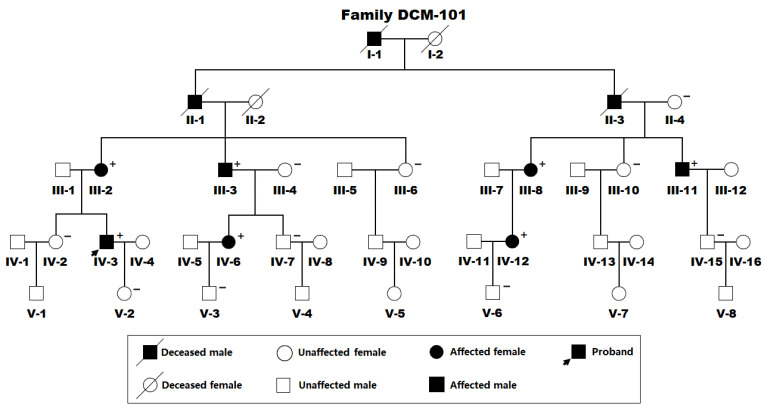
The pedigree of the studied family suffering from dilated cardiomyopathy. Family members are identified by generation and number (using a Roman and an Arabic numeral, respectively). “+” denotes a carrier of the detected *ETS1* mutation in a heterogeneous status; “−” means a non-carrier.

For the current investigation, as illustrated in Figure 1, a 42-member pedigree affected with DCM spanning five generations was identified as Family DCM-101 from the Chinese Han-ethnicity population. In this 42-member family with DCM, there were 37 living members, encompassing 19 male and 18 female family members. The proband of Family DCM-101 (IV-3) was first diagnosed with DCM at the age of 41 years. During the proband’s clinical follow-up, he experienced progressive fatigue and dyspnea on exertion, left ventricular dilation and systolic dysfunction on echocardiograms, and he received pharmacologic therapies (intravenous injections, infusions, and oral medications) for chronic cardiac failure, encompassing diuretics (furosemide and spironolactone), angiotensin-converting enzyme inhibitor (captopril), and a β-adrenergic receptor antagonist (metoprolol). His grandfather (II-1) and two other relatives (II-3 and I-1) had a past medical history of DCM and died of progressive congestive heart failure attributable to DCM. His grandmother (II-2) and another relative (I-2) had no medical history of DCM and died of a cerebral stroke.

Additionally, in Family DCM-101, all 10 affected members were diagnosed with DCM based on clinical symptoms, physical signs, and echocardiographic findings, while all unaffected members denied a medical history of DCM, with no echocardiographic abnormality. No individuals from Family DCM-101 had known environmental pathogenic factors predisposing to DCM, encompassing viral myocarditis, coronary heart disease, autoimmune disorders, rheumatic heart disease, and arterial hypertension. A genetic evaluation of the family (Family DCM-101) revealed that DCM was inherited as an autosomal-dominant trait in the whole pedigree, with complete penetrance. Notably, all 10 affected members also suffered mild facial dysmorphisms, including a high forehead, a broad nasal bridge with bulbous tip, and dysplastic little ears. Furthermore, in addition to DCM, three family members (III-8, III-11, and IV-12) also had atrial fibrillation, and one family member (IV-6) also had a minor membranous ventricular septal defect (VSD) that closed spontaneously within the first year of her life. The demographic and baseline phenotypic characteristics of the living family members with DCM from Family DCM-101 are outlined in Table 1.

### 3.2. Discovery of a New DCM-Causative Mutation in ETS1

A WES assay was completed in four DCM-affected members (III-2, III-8, IV-3, and IV-12 from Family DCM-101) and three non-DCM members (IV-2, V-2, and V-6 from Family DCM-101). A mean of 15,209 nonsynonymous genetic variations (ranging from 14,917 to 17,536) per family member passed filtering on the likely inheritance modes (the recessive genetic transmission mode was also encompassed during screening for potential causative genetic variations), of which 11 heterozygous missense and nonsense genetic variations passed filtering by ANNOVAR, with a minor allele frequency of <0.01%, and were carried by all the four DCM-affected members (III-2, III-8, IV-3, and IV-12 from Family DCM-101) who had experienced WES analysis, as given in Table 2.

Further Sanger sequencing assays of these genetic variations in all the family members available from Family DCM-101 revealed that only the heterozygous nonsense mutation of chr11:128,355,998T>G (GRCh37.p13/hg19: NC_000011.9), equivalent to chr11:128,560,220T>G (GRCh38.p14/hg38: NC_000011.10), or NM_005238.4:c.447T>G;p.(Tyr149*) in the erythroblast transformation-specific 1 (*ETS1*) gene, was verified to co-segregate with the DCM phenotype in the entire pedigree, i.e., present in all DCM members but absent in all unaffected members of Family DCM-101. However, Sanger sequencing assays of the other 10 genetic variations demonstrated that none was in co-segregation with the DCM phenotype in the whole family, suggesting that none of these 10 genetic variations is likely to underlie DCM in this family. In addition, a Sanger sequencing assay of the *ETS1* gene in a total of 276 unrelated control people revealed no pathogenic mutations. The detected *ETS1* mutation was absent in dbSNP (accessed on 20 June 2025) and gnomAD (accessed on 20 June 2025), suggesting a novel *ETS1* mutation. The primers designed to specifically amplify the *ETS1* gene (including the whole set of coding exons and splicing boundaries) by PCR are provided in Table 3.

**Figure 2:**
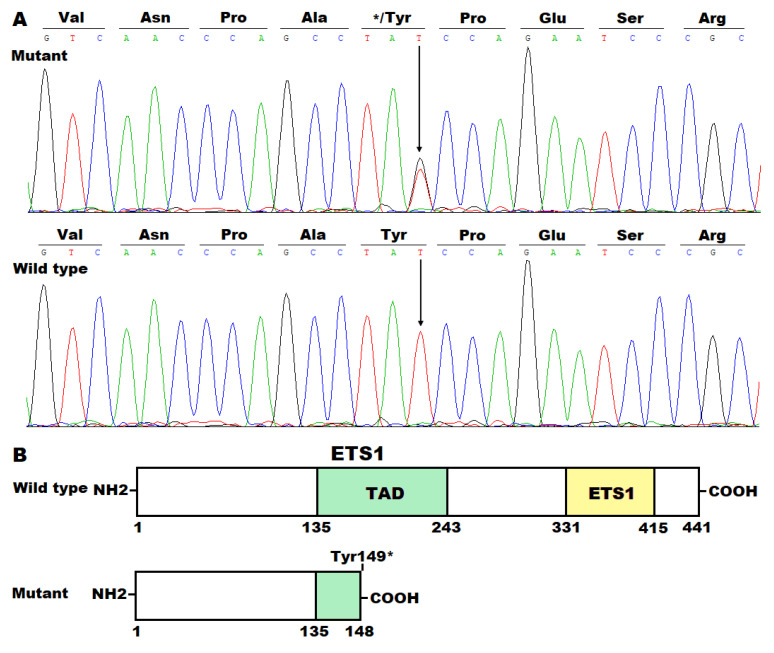
A novel *ETS1* mutation identified to account for familial dilated cardiomyopathy. (**A**) Sequence chromatograms demonstrate the heterogeneous mutant *ETS1* alleles (T/G) in the affected proband (mutant) and wild-type *ETS1* alleles (T/T) in an unaffected individual (wild type). An arrow points to where the mutation occurs in the proband’s *ETS1* allele. (**B**) The schematic diagrams signify the structural domains of the ETS1 protein. ETS1, erythroblast transformation-specific 1; TAD, transcription activation domain.

The DNA sequence chromatograms illustrating the heterozygous mutant *ETS1* alleles (T/G) as well as wild-type *ETS1* alleles (T/T) are provided in Figure 2A. The schematic diagrams delineating the functional motifs of the ETS1 proteins are given in Figure 2B.

### 3.3. Functional Loss of Tyr149*-Mutant ETS1 in Transactivating CLDN5

**Figure 3:**
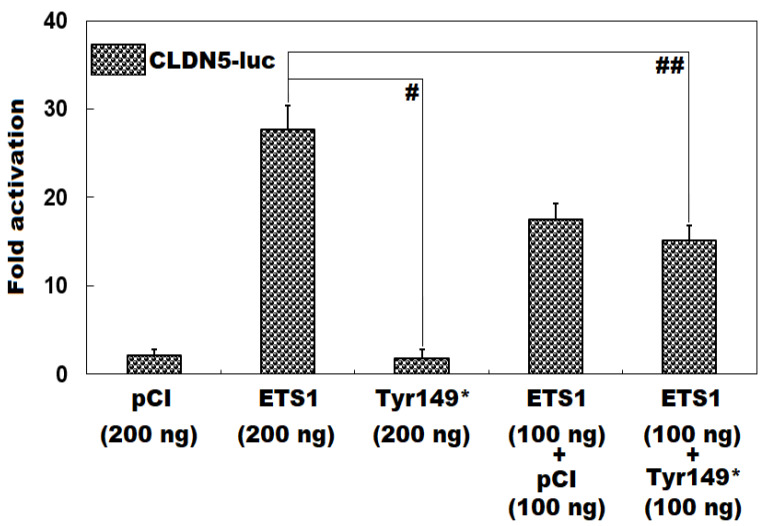
Functional loss of Tyr149*-mutant ETS1 in transactivation of *CLDN5*. A dual reporter gene assay of the transactivation of the *CLDN5* promoter-induced luciferase expression in cultivated HeLa cells by human wild-type ETS1-pCI-neo (ETS1) or Tyr149*-mutant ETS1-pCI-neo (Tyr149*), alone or together, showed that the Tyr149*-mutant ETS1 protein lost transactivation function. For each expression vector utilized, three independent cellular transfections were conducted and analyzed in triplicate. Herein, # and ## indicate *p* < 0.001 and *p* < 0.005.

As exhibited in Figure 3, in HeLa cells routinely cultivated in vitro and transiently transfected with various eukaryotic expression plasmids, the wild-type ETS1-pCI-neo plasmid and Tyr149*-mutant ETS1-pCI-neo plasmid induced transcriptional activation of the *CLDN5* promoter by ~28-fold and ~2-fold, respectively (Tyr149*-mutant ETS1-pCI-neo vs. wild-type ETS1-pCI-neo: *t* = 15.8273; *p* = 0.0001). When the wild-type ETS1-pCI-neo and Tyr149*-mutant ETS1-pCI-neo plasmids were co-transfected, the elicited transactivation activity was ~15-fold (wild-type ETS1-pCI-neo vs. wild-type ETS1-pCI-neo + Tyr149*-mutant ETS1-pCI-neo: *t* = 6.9335; *p* = 0.0023). When the comparisons among multiple groups were implemented, similar resultant data were yielded (F = 127.82, *p* = 1.534 × 10^−8^). Multiple-group comparisons were performed between 200 ng of empty pCI-neo plasmid and 200 ng of wild-type ETS1-pCI-neo plasmid (*t* = 25.59, *p* < 0.0001), between 200 ng of empty pCI-neo plasmid and 200 ng of Tyr149*-mutant ETS1-pCI-neo plasmid (*t* = 0.3533, *p* = 0.9989), between 200 ng of empty pCI-neo plasmid and 100 ng of empty pCI-neo plasmid + 100 ng of wild-type ETS1-pCI-neo plasmid (*t* = 15.4233, *p* < 0.0001), between 200 ng of empty pCI-neo plasmid and 100 ng of wild-type ETS1-pCI-neo plasmid + 100 ng of Tyr149*-mutant ETS1-pCI-neo plasmid (*t* = 13.04, *p* < 0.0001), between 200 ng of wild-type ETS1-pCI-neo plasmid and 200 ng of Tyr149*-mutant ETS1-pCI-neo plasmid (*t* = 25.9433, *p* < 0.0001), between 200 ng of wild-type ETS1-pCI-neo plasmid and 100 ng of empty pCI-neo plasmid + 100 ng of wild-type ETS1-pCI-neo plasmid (*t* = 10.1667, *p* = 0.0002), between 200 ng of wild-type ETS1-pCI-neo plasmid and 100 ng of wild-type ETS1-pCI-neo plasmid + 100 ng of Tyr149*-mutant ETS1-pCI-neo plasmid (*t* = 12.55, *p* < 0.0001), between 200 ng of Tyr149*-mutant ETS1-pCI-neo plasmid and 100 ng of empty pCI-neo plasmid + 100 ng of wild-type ETS1-pCI-neo plasmid (*t* = 15.7767, *p* < 0.0001), between 200 ng of Tyr149*-mutant ETS1-pCI-neo plasmid and 100 ng of wild-type ETS1-pCI-neo plasmid + 100 ng of Tyr149*-mutant ETS1-pCI-neo plasmid (*t* = 13.3933, *p* < 0.0001), and between 100 ng of empty pCI-neo plasmid + 100 ng of wild-type ETS1-pCI-neo plasmid and 100 ng of wild-type ETS1-pCI-neo plasmid + 100 ng of Tyr149*-mutant ETS1-pCI-neo plasmid (*t* = 2.3833, *p* = 0.4603).

### 3.4. Inability of Tyr149*-Mutant ETS1 to Transcriptionally Activate ALK1

**Figure 4:**
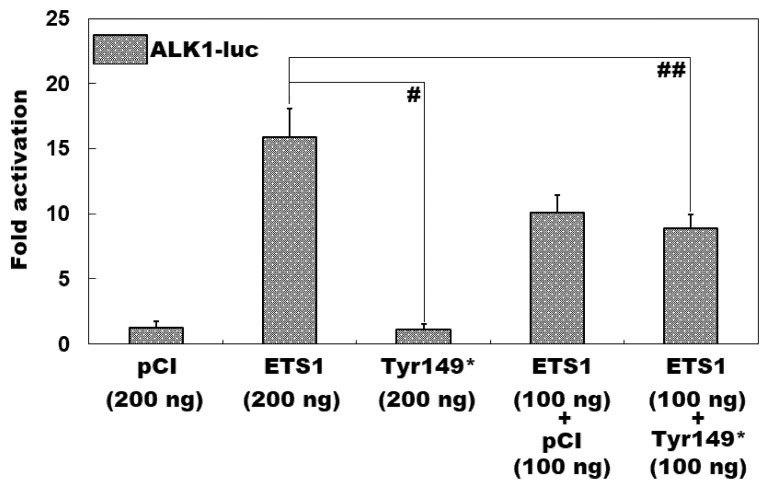
Inability of Tyr149*-mutant ETS1 to transactivate *ALK1*. A dual reporter gene assay of the transactivation of the *ALK1* promoter-driven luciferase expression in HeLa cells cultured in vitro by human wild-type ETS1-pCI-neo (ETS1) or Tyr149*-mutant ETS1-pCI-neo (Tyr149*), singly or together, revealed that the Tyr149*-mutant ETS1 protein had no transcriptional function. For each plasmid utilized, three independent cellular transfections were performed and assayed in triplicate. Herein, # indicates *p* < 0.001 and ## indicates *p* < 0.01.

As displayed in Figure 4, in HeLa cells routinely cultured in vitro and transiently transfected with various eukaryotic expression plasmids, the wild-type ETS1-pCI-neo plasmid and Tyr149*-mutant ETS1-pCI-neo plasmid induced transcriptional activation of the *ALK1* promoter by ~16-fold and ~1-fold, respectively (Tyr149*-mutant ETS1-pCI-neo vs. wild-type ETS1-pCI-neo: *t* = 11.3841; *p* = 0.0003). When the wild-type ETS1-pCI-neo and Tyr149*-mutant ETS1-pCI-neo plasmids were co-transfected, the elicited transactivation activity was ~9-fold (wild-type ETS1-pCI-neo vs. wild-type ETS1-pCI-neo + Tyr149*-mutant ETS1-pCI-neo: *t* = 4.9510; *p* = 0.0078). When the comparisons among multiple groups were implemented, similar statistical data were generated (F = 71.745, *p* = 2.524 × 10^−7^). Multiple-group comparisons were performed between 200 ng of empty pCI-neo plasmid and 200 ng of wild-type ETS1-pCI-neo plasmid (*t* = 14.6167, *p* < 0.0001), between 200 ng of empty pCI-neo plasmid and 200 ng of Tyr149*-mutant ETS1-pCI-neo plasmid (*t* = 0.1333, *p* = 0.9999), between 200 ng of empty pCI-neo plasmid and 100 ng of empty pCI-neo plasmid + 100 ng of wild-type ETS1-pCI-neo plasmid (*t* = 8.8067, *p* < 0.0001), between 200 ng of empty pCI-neo plasmid and 100 ng of wild-type ETS1-pCI-neo plasmid + 100 ng of Tyr149*-mutant ETS1-pCI-neo plasmid (*t* = 7.64, *p* = 0.0002), between 200 ng of wild-type ETS1-pCI-neo plasmid and 200 ng of Tyr149*-mutant ETS1-pCI-neo plasmid (*t* = 14.75, *p* < 0.0001), between 200 ng of wild-type ETS1-pCI-neo plasmid and 100 ng of empty pCI-neo plasmid + 100 ng of wild-type ETS1-pCI-neo plasmid (*t* = 5.81, *p* = 0.0018), between 200 ng of wild-type ETS1-pCI-neo plasmid and 100 ng of wild-type ETS1-pCI-neo plasmid + 100 ng of Tyr149*-mutant ETS1-pCI-neo plasmid (*t* = 6.9767, *p* = 0.0004), between 200 ng of Tyr149*-mutant ETS1-pCI-neo plasmid and 100 ng of empty pCI-neo plasmid + 100 ng of wild-type ETS1-pCI-neo plasmid (*t* = 8.94, *p* < 0.0001), between 200 ng of Tyr149*-mutant ETS1-pCI-neo plasmid and 100 ng of wild-type ETS1-pCI-neo plasmid + 100 ng of Tyr149*-mutant ETS1-pCI-neo plasmid (*t* = 7.7733, *p* = 0.0002), and between 100 ng of empty pCI-neo plasmid + 100 ng of wild-type ETS1-pCI-neo plasmid and 100 ng of wild-type ETS1-pCI-neo plasmid + 100 ng of Tyr149*-mutant ETS1-pCI-neo plasmid (*t* = 1.1667, *p* = 0.7976).

## 4. Discussion

In the present investigation, a five-generation pedigree of 42 members affected with DCM was enrolled. By means of a WES-based bioinformatics assay in the available family members, a heterozygous truncating *ETS1* mutation, i.e., NM_005238.4:c.447T>G;p.(Tyr149*), was discovered and verified via a Sanger sequencing assay to co-segregate with DCM in the entire family. This *ETS1* mutation was neither observed in the 552 reference human chromosomes from the Chinese Han population nor found in the population genetics databases of dbSNP and gnomAD. Quantitative biochemical measurement based on dual reporter genes demonstrated that Tyr149*-mutant ETS1 failed to transactivate the expression of *CLDN5* (encoding claudin 5, an integral tight junction protein component) and *ALK1* (coding for activin receptor-like kinase 1), two genes pivotal for proper cardiovascular embryonic development and postnatal structural remodeling [75,76,77,78,79,80,81,82,83,84,85]. These results cumulatively indicate that genetically compromised *ETS1* gives rise to the development of DCM in humans.

In humans, *ETS1* (also known as *ETS-1*, *TPL*-*1*, *c*-*ETS-1*, *c*-*ETS*, *EWSR2*, *P54*, or *ETS*) is mapped on chromosome 11q24.3; it contains 8 coding exons and encodes an integral tight junction protein with 441 amino acids, the founding member of the ETS-domain transcription factor family, which is defined by the existence of an evolutionarily conserved ETS DNA-binding motif that recognizes and binds to the core consensus DNA sequence of GGAA/T (a unique ETS-binding site) in the promoters/enhancers of target genes [86,87,88,89]. The ETS family of transcription factors currently comprises 28 members in humans and 27 members in mice, and they function as transcriptional regulators (activators or repressors) of numerous downstream target genes that are involved in an extensive assortment of important biological processes including cellular growth, proliferation, differentiation, migration, apoptosis, senescence, and death, hence playing crucial roles in embryonic organogenesis, tissue homeostasis, and the progression of diseases, including arteriosclerosis, autoimmune diseases, allergies, and tumors [86,87,88,89]. Among the ETS family, ETS1 is characterized by its broad expression, especially by its ample expression in the heart at all stages from fetuses to adult humans [90,91,92] and multiple species of animals [93,94,95,96,97,98]. Moreover, ETS1 has been validated to have a potent transcriptional regulation function, playing a key role in normal cardiovascular development and postnatal structural remodeling, and in mice, deletion of *Ets1* leads to various cardiovascular developmental abnormalities, including VSD, double-outlet right ventricle (DORV), and ventricular noncompaction [93,94,95,96,97]. ETS1 contains two functionally critical structural domains, including a transcriptional activation domain (TAD) and an ETS domain responsible for recognizing and binding to the promoters/enhancers of target genes [86,87,88,89]. In the present research, we identified a novel heterozygous *ETS1* mutation, NM_005238.4:c.447T>G;p.(Tyr149*), to co-segregate with DCM in the whole family. This mutation was anticipated to create a truncated ETS1 protein (Tyr149*), with the entire ETS domain along with a larger part of the TAD domain lost, hence, presumably, leading to a loss of transcriptional regulation function, which was confirmed by biochemical assays of its two representative target genes, *CLDN5* and *ALK1*. These results support the hypothesis that the haploinsufficiency of *ETS1* predisposes individuals to DCM.

It may be attributed to improper embryonic development and structural remodeling of the heart that *ETS1* haploinsufficiency contributes to DCM. In mice (with a C57BL/6 genetic background), homozygous knockout of *Ets1* led to almost complete perinatal lethality because of profound cardiovascular developmental defects, including a membranous VSD and an anomalous cartilage nodule in the heart [93]. Ye and colleagues [94] reported that in mice (with a C57/B6 background), ETS1 was highly expressed in the cardiac neural crest, endocardium, and vascular endothelium early during embryogenesis, and targeted disruption of *Ets1* caused a large membranous VSD and a bifid cardiac apex, as well as a non-apex-forming left ventricle, a hallmark of hypoplastic left heart syndrome (HLHS). Lin et al. [95] reported that deletion of *Ets1* in mice resulted in a cardiovascular developmental defect resembling a DORV in humans. Furthermore, endothelial-specific knockout of *Ets1* in mice led to ventricular noncompaction and coronary vascular developmental anomaly, which reproduced the phenotype caused by global deletion of *Ets1* [96]. Notably, Endothelial-specific ablation of *Ets1* downregulated the expression levels of multiple target genes, including *Alk1* and *Cldn5* [96]. Recently, Nie and Bronner [98] demonstrated that in *Xenopus*, ETS1 was abundantly expressed in the cardiac neural crest and mesoderm during embryogenesis, and knockdown of *Ets1* in the cardiac mesoderm gave rise to an HLHS-like ventricle, which was characteristic of an abundance of cardiomyocytes and a loss of endocardial cells, in line with a key role for ETS1 in mediating cellular fate determination between cardiac myocytes and endocardial cells [97,98]. Zhou and co-workers [99] reported that in the murine embryonic heart, the cardiomyocyte- and endothelial-restricted *Gata4*-occupied genomic regions were enriched for *Ets1* and *Nkx2-5* motifs, respectively, and both ETS1 and NKX2-5 transcription factors interacted with GATA4 to regulate heart development. Of note, both GATA4 and NKX2-5 have been causally linked to human DCM [100,101,102,103,104]. Additionally, in an experimental mouse model with autoimmune myocarditis, treatment with spironolactone (an aldosterone receptor antagonist) significantly alleviated myocardial hypertrophy, improved cardiac function, and diminished myocardial fibrosis via inhibition of ETS1 [105]. In rodent/rat and cellular models with myocardial ischemia/reperfusion (hypoxia/reoxygenation) injury, knockout of *Kdm3a* (coding for histone demethylase KDM3A) exacerbated myocardial injury and cardiac dysfunction, and deteriorated mitochondrial apoptosis and inflammation; conversely, overexpression of KDM3A could ameliorate such alterations by promoting ETS1 expression [106]. In chicken embryos, copious expression of ETS1 was observed in the heart and endothelial cells of blood vessels [107], and knockdown of *Ets1* hampered the development of the myocardium and coronary system, resulting in a defect in myocardial perfusion [108]. In addition, in cultured ventricular myocytes from chick embryos, ETS1 was shown to transactivate the expression of inducible nitric oxide synthase, presumably via interaction with NF-kappaB [109]. In human embryonic stem cells, ETS1 was identified by single-cell reconstruction of differentiation trajectory to be a pivotal factor responsible for cardiac lineage commitment from a pluripotent status [110]. Taken together, these results highlight the crucial roles of ETS1 in embryonic cardiogenesis and postnatal cardiac remodeling, suggesting that an ETS1 loss-of-function mutation is a molecular defect underlying DCM in humans.

Previously, in humans, chromosome 11q terminal deletions (11q−) containing *ETS1* and *ETS1* variations have been implicated in the pathogenesis of Jacobsen syndrome (JS), a rare chromosomal/genetic disorder characterized by a wide spectrum of phenotypes with varying degrees of severity, including congenital heart defects, facial dysmorphisms, intellectual disability, autism and attention deficit, immunodeficiency, genitourinary tract deformities, growth restriction, and thrombocytopenia [111,112,113]. By comparative genomic hybridization and fluorescence in situ hybridization analyses in five patients from two families with JS, Conrad and partners [111] detected inherited interstitial deletions in the chromosomal region of 11q24.2-q24.3, the smallest ever reported at the JS locus. Specifically, in Family A, a 700 kb interstitial chromosomal deletion of 11q24.3 containing three genes, *ETS1*, *FLI1*, and *SENCR*, was observed; in Family B, a 1.5 Mb interstitial chromosomal deletion of 11q24.2-q24.3 containing eight genes, *ARHGAP32*, *ETS1*, *TP53AIP1*, *FLI1*, *KCNJ1*, *C11orf45*, *SENCR*, and *KCNJ5*, was observed [111]. Of the two JS families, all affected members had *ETS1* haploinsufficiency and manifested such clinical characteristics of JS as developmental malformations (two patients from Family A had minor VSD), immune deficiency, and thrombocytopenia, but had normal cognitive development, consistent with an incomplete penetrance of JS [111]. Grossfeld et al. [112] conducted a prospective study of 110 JS patients with the terminal deletion of chromosome 11q diagnosed by karyotypic analysis, of whom 93 JS patients underwent a comprehensive cardiovascular assessment. As a result, 56% (52/93) of JS patients had severe cardiovascular developmental defects, of which the majority required surgical intervention [112]. In total, two-thirds of the JS patients had a variety of congenital heart defects, including membranous VSD, secundum atrial septal defects, aortic coarctation/stenosis, patent ductus arteriosus, atrioventricular canal defects, mitral valve stenosis, double-outlet right ventricle, bicuspid aortic valve, transposition of the great arteries, aberrant right subclavian artery, dextrocardia, a left-sided superior vena cava, and hypoplastic left heart syndrome [112]. By means of a genome-wide single-nucleotide polymorphism array and whole-exome sequencing assays in 538 trios with congenital heart defects, Glessner et al. [113] identified a de novo *ETS1* frameshift variant (c.1044_1049delCAAGGAinsTT or p.Lys349SerfsX2) in one patient with congenital heart malformations (hypoplastic left heart syndrome). Additionally, the integrated data implied that *ETS1* was the causal gene altered by chromosome 11q24.2-q25 deletions in JS [113]. In the current study, a novel *ETS1* loss-of-function variant (c.447T>G or p.Tyr149*) was causally linked to familial DCM, hence expanding the phenotypic spectrum of *ETS1* variations. Notably, there is an urgent need to investigate whether DCM occurs in small or large animals after knockout of *Ets1* or knock-in of the DCM-causing *Ets1* mutation, which is our future research direction. Additionally, the preparation and characterization of induced pluripotent stem cell-derived cardiomyocytes from patients and healthy individuals of the same family with DCM or patient-derived fibroblasts will help better understand the pathogenic effect of the mutated *ETS1* gene on cardiac tissue [114,115,116,117].

To date, pathogenic variations in >250 genes have been implicated in the development of human DCM [1,2,33,34,35,36,37,38,39,40,41,42,43,44,45,46,47,48,49,50,51,52,53,54,55,56,57,58,59,60,61]. Nevertheless, there are still clear reasons why it is necessary to search for a new causative gene for DCM. First, due to substantial genetic heterogeneity, these known causative genes can merely explain 25–50% of all DCM cases, and the genetic determinants responsible for DCM in 50–75% of cases remain elusive, highlighting the need to identify new DCM-causative genes [1,2,25]. Second, the discovery of the DCM-causing mutation is of important clinical significance, since early interventions for DCM have been proven to be valuable in reducing morbidity and mortality [118]. Third, the identification of DCM-causing genes has a direct clinical impact on the families as well as the population involved, which immediately provides possibilities for antenatal/presymptomatic prevention of DCM through genetic screening of mutation carriers and prenatal testing, and ensures healthy family members reproduce healthy children [118]. Finally, the discovery of causative genes holds the potential to add more insight into the etiology and pathophysiology of diseases, and, therefore, potential improved therapy [118].

## 5. Conclusions

This investigation unveils *ETS1* as a new gene accountable for DCM in humans, thereby offering novel insights into the pathogenesis of the disease and holding translational potential for future prophylactic and therapeutic strategies.

## Figures and Tables

**Table 1:** Demographic and baseline phenotypic characteristics of the affected pedigree members alive from Family DCM-101.

Individuals (Family DCM-101)	Sexes	Ages (Years)	Cardiac Phenotypes	LVEDD (mm)	LVESD (mm)	LVEF (%)	LVFS (%)
III-2	Female	68	DCM	71	62	33	18
III-3	Male	65	DCM	76	65	30	14
III-8	Female	63	DCM, AF	70	60	27	13
III-11	Male	57	DCM, AF	69	58	32	16
IV-3	Male	45	DCM	58	48	36	18
IV-6	Female	40	DCM, VSD	64	52	38	19
IV-12	Female	38	DCM, AF	60	50	40	20

DCM, dilated cardiomyopathy; LVEF, left ventricular ejection fraction; AF, atrial fibrillation; LVESD, left ventricular end-systolic diameter; VSD, ventricular septal defect; LVEDD, left ventricular end-diastolic diameter; LVFS, left ventricular fractional shortening.

**Table 2:** Candidate genetic variations for dilated cardiomyopathy revealed by an exome-wide sequencing assay.

Chr.	Position (hg19)	Ref.	Alt.	Gene	Variation
1	92,184,903	G	A	*TGFBR3*	NM_003243.5: c.1532G>A; p.(Trp511*)
2	141,777,525	C	G	*LRP1B*	NM_018557.3: c.1936C>G; p.(His646Asp)
3	71,096,210	C	G	*FOXP1*	NM_032682.6: c.547C>G; p.(Gln183Glu)
4	157,693,868	A	G	*PDGFC*	NM_016205.3: c.673A>G; p.(Lys225Glu)
6	38,029,545	G	C	*ZFAND3*	NM_021943.3: c.289G>C; p.(Glu97Gln)
7	133,689,816	C	T	*EXOC4*	NM_021807.4: c.2500C>T; p.(Gln834*)
11	128,355,998	T	G	*ETS1*	NM_005238.4: c.447T>G; p.(Tyr149*)
12	66,773,093	G	T	*GRIP1*	NM_021150.4: c.2432G>T; p.(Ser811Ile)
15	86,266,500	C	G	*AKAP13*	NM_006738.6: c.6706C>G; p.(Leu2236Val)
16	49,671,508	T	C	*ZNF423*	NM_015069.5: c.1555T>C; p.(Cys519Arg)
18	34,092,506	G	A	*FHOD3*	NM_025135.5: c.511G>A; p.(Ala171Thr)

Chr., chromosome; Ref., reference; Alt., alteration.

**Table 3:** Primers to specifically amplify the coding exons and splicing boundaries of the *ETS1* gene.

Coding Exons	Forward Primers (5′→3′)	Reverse Primers (5′→3′)	Amplicon Sizes (bp)
1	AAAGTCGGATTTCCCCCGTC	AGGGTTCTCGCTCATTTGGG	515
2	CTGGTGCTCTGGTGAGGATG	CTGGTGCTCTGGTGAGGATG	486
3	TTGGCTGGCAGTAGTGACCT	GGCACATTCCCACATACGTC	571
4	TCTTTAGACAAGGCCACAGTCA	TGGCCATCGTAGTGCACAGT	568
5	CATAGGGCCCTGTAGATGGTG	GGACTTGGCTAAAACACCACG	514
6	CCCAAACGCTACTCCGACAG	AAAGGGAGTCTCTCGTCGTC	664
7	GAAGAGGGATGTGGGAGTGC	CACAGGAGGCAGCTCTATGG	491
8	TTGTGACTCCCATCCTTGCC	GCTTTCCTTTCCCAACTGCG	609

## Data Availability

The data in favor of the discovery in this study are available upon reasonable request.
